# Personal

**Published:** 1903-09

**Authors:** 


### PERSONAL.

Dr. Alexander Hugh Ferguson, of Chicago, delivered the address in surgery at the recent meeting of the Canadian Medical Association, held at London, Ont.

Dr. Roswell Park, of Buffalo, was grievously ill of erysipelas for some weeks during the early summer, but has recovered and resumed his professional work

Dr. Frank W. McGuire, of Buffalo, was appointed gynecologist to the Buffalo German Hospital at the last meeting of the staff, vice Dr. H. E. Hayd, appointed surgeon to the hospital.

Dr. Irving M. Snow and his mother, Mrs. Julia F. Snow, of Buffalo, sailed August 5, 1903, for Liverpool, to enjoy a short European tour.

Dr. Lillian Craig Randall, of Riverside Hospital, Buffalo, and Miss Edna Craig Randall, sailed July 8, 1903, on the White Star liner "Teutonic" for Liverpool.

Dr. Frederick Preiss,was given a dinner at the Genesee Hotel recently by a number of his friends, to commemorate his return from Europe. Those present besides the guest of honor were Drs. R. L. Banta, J. C. Thompson, George H. McMichael, and William Preiss; and Henry Zipp, William Larkins, Dan T. Murray, Walter C. Kelly, Edwin Leslie, A. E. Richmond, E. W. Mills, and John Murphy.

---

Dr. Matthew D. Mann, of Buffalo, delivered the address in gynecology at the annual meeting of the Canadian Medical Association, held at London, Ont., August 25-28, 1903.

---

Dr. Joseph M. Mathews, of Louisville, according to announcement in the *Louisville Monthly Journal of Medicine and Surgery,* has resigned as professor of surgery and clinical lecturer in the Hospital College of Medicine and Surgery. Dr. Mathews will, it is stated, devote his time principally to literary work, to travel and to his practice.

---

Dr. Charles A. L. Reed, of Cincinnati, accompanied by his family, sailed for Naples, by the Favre line, August 25, 1903. He will leave his family at Florence, visit Paris and London, and return by the "Teutonic," due to arrive at New York, October 21.

---

Dr. L. S. McMurtry, of Louisville, paid a visit to Buffalo and Niagara Falls during the latter days of August. He spoke enthusiastically of the progress Buffalo is making, as indicated by the many improvements which have taken place since the Pan-American season.

